# Current Models for Predicting Drug-induced Cholestasis: The Role of Hepatobiliary Transport System

**DOI:** 10.22037/ijpr.2020.113362.14254

**Published:** 2021

**Authors:** Farahnaz Jazaeri, Mohammad Sheibani, Sadaf Nezamoleslami, Leila Moezi, Ahmad-Reza Dehpour

**Affiliations:** a *Experimental Medicine Research Center, Tehran University of Medical Sciences, Tehran, Iran. *; b *Department of Pharmacology, School of Medicine, Tehran University of Medical Sciences, Tehran, Iran.*; c *Department of Pharmacology, School of Medicine, Iran University of Medical Sciences, Tehran, Iran. *; d *Department of Pharmacology, School of Medicine, Shiraz University of Medical Sciences, Shiraz, Iran.*; e *F. J. and M. Sh. contributed equally to this work.*

**Keywords:** Drug, Cholestasis, In-vivo, In-vitro, Models, Transport system

## Abstract

Drug-induced cholestasis is the main type of liver disorder accompanied by high morbidity and mortality. Evidence for the role of hepatobiliary pumps in the cholestasis patho-mechanism is constantly increasing. Recognition of the interactions of chemical agents with these transporters at the initial phases of drug discovery can help develop new drug candidates with low cholestasis potential. This review delivers an outline of the role of these transport proteins in bile creation. It addresses the pathophysiological mechanism for drug-induced cholestasis. *In-vitro *models, including cell-based and membrane-based approaches and *In-vivo *models such as genetic knockout animals, are considered. The benefits and restrictions of each model are discussed in this review. Current understandings into the cellular and molecular process that control the activity of hepatobiliary pumps have directed to a better understanding of the pathophysiology of drug-induced cholestasis. A combination of *in-vitro *monitoring for transport interaction, *in-silico *predicting systems, and consideration of and metabolic and physicochemical properties must cause more effective monitoring of possible liver problems.

## Introduction

The liver is the main location for biological processes and the deployment of drugs. 

Owing to its intermediate role in interrelating the gastrointestinal and blood circulation systems, the liver removes xenobiotics before their entrance into portal blood circulation. 

Liver anatomical ensure passage of many of drugs into hepatic tissues, resulting in accumulation of these within hepatocytes. Drugs are considered the most common etiology underlying acute liver insufficiency known as drug-induced liver injury (DILI). This phenomenon itself is responsible for unsuccess rate of new drugs development. Around 2-5% of jaundices requiring hospitalization are related to DILI, while DILI is also the major culprit of 10% of hepatitis outbreaks in adulthood. In addition, as high as 40% of hepatitis in individuals older than 50 years old have been associated with DILI (1). Furthermore, most patients with DILI had an acute type of disease (74 percent) (2).

DILI can be an asymptomatic clinical entity with only slight alternations in biochemical parameters. However, it is not uncommon for DILI to present with acute jaundice reminiscing acute viral hepatitis (3, 4). Classification of DILI relies on both the degree of liver damage and the extent of biochemical derangements. Accordingly, DILI can be categorized as either hepatocellular, cholestatic, or mixed type damage (5). Cholestatic and hepatocellular injury are two of the most severe manifestations of DILI, responsible for nearly 50% of cases of all drug-related hepatic damage in some reports (6). The main feature of the cholestatic is elevated levels of alkaline phosphatase (ALP), glutamyltranspeptidase, and conjugated bilirubin blood levels. Characteristics and exact etiological and pathophysiological features of hepatocellular DILI are not well recognized. On the other hand, it is supposed that blockage of polar hepatobiliary transporters by either drugs or their metabolites constitute the major pathophysiological finding in cholestatic DILI (7).

Transporters located at the basolateral surface of the hepatobiliary system are the entrance gate for drugs into hepatic tissue. In contrast, those transporters expressed at the apical or canalicular side mainly clear drugs or their metabolites from the hepatobiliary system and release bile components. The abnormal function of these transporters may lead to bile deposition and finally cholestatic liver cell damage. Furthermore, reabsorption and shunting of ductular bile components to the liver can change the bile composition and accumulate them in the liver. 

Currently, diagnosis of drug-induced cholestasis (DIC) creation is hindered by a lack of appropriate *in-vitro *or *In-vivo *screening tests. Establishing DIC may be difficult in phase III clinical trials. Following the administration of some approved drugs, there have been reports of severe liver toxicity leading to death. Because of these observations, such drugs received black-box warnings or even been withdrawn from the market, especially for those associated with mortalities. In this regard, Troglitazone and Bromfenac are two drugs that have been withdrawn, and Bosentan and Diclofenac have received the warnings. So, clinical detection of drug-induced cholestasis remains a challenge during the development of a drug. Antibiotics, antifungals, psychotropic drugs and oral contraceptives are common drugs associated with cholestatic liver injury (8). Regarding these findings, we explored the models for predicting potential cholestatic effects of drugs. We also resume the hepatic mechanisms responsible for drug influx and efflux which participate in bile formation. 

**Physiology of bile formation**

Bile synthesis acts as the main gateway for the excretion of various endogenous and exogenous compounds such as bile acids and salts, lipids like cholesterol and phosphatidylcholine, organic anions such as bilirubin and drugs (9). Elimination of bile from the liver takes place through the bile ducts and gallbladder and finally opens into the small intestine (10). It helps and promotes fat metabolism and thus triggers the absorption of dietary fatty acids and vitamins like vit-K, vit-D and vit-E (11). The major driving force to initiate bile flow is the osmotic pressure generated by Bile salts; however, other mechanisms also contribute to such phenomena (12). Biliary components are transported from blood by a sinusoidal intracellular transport system into the hepatocyte (with or without metabolic modifications) or through canaliculi secretion which works against the concentration gradients and is energy-dependent transport (13, 14). The water concentration along the canaliculi is the major determinant for the bile flow and further changes in the composition of bile takes place along the biliary trunk (15). In addition, the bile salts is transported along the last portion of ileum for recycling through enterohepatic pathway (13, 16). Thus this phenomenon helps (17) to recycle the bile salts for reuse after its first release. Cholestasis is a condition that arises when there is a homeostatic imbalance in the metabolism or recycling of bile acid. Bile acids may become toxic if accumulated in high concentration within the hepatocytes and may cause liver damage (18, 19).

**Molecular basis of hepatobiliary transport**

**Figure 1:**
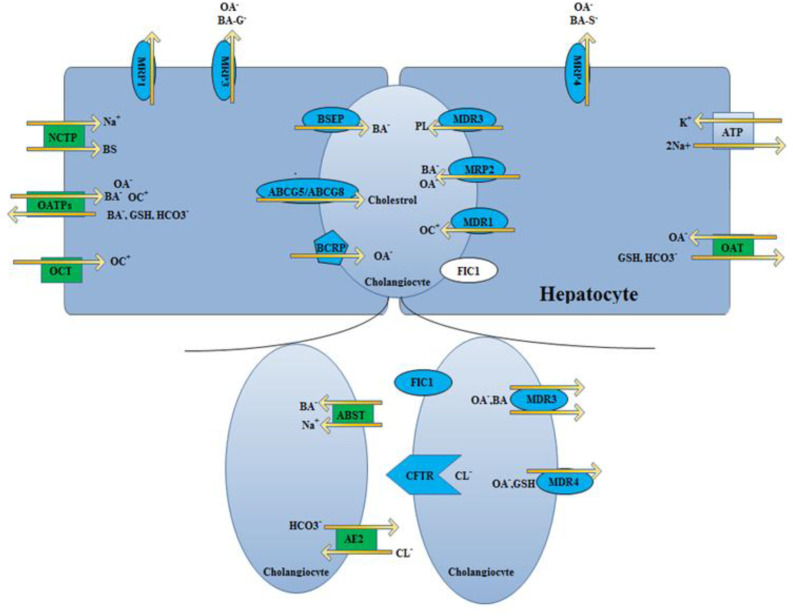
Hepatobiliary transport proteins involved in bile formation. Transporters imprinted in green correspond to proteins belonging to the solute carrier (SLC) family, whereas those imprinted in blue belong to the ATP-binding cassette (ABC) transporter family. The 'half ABC transporters' are depicted as polygones. The cystic fibrosis transmembrane conductance regulator (CFTR), which functions as a Cl- channel, is shown as a pentagon arrow. We have to know that channels are not transporters. The hepatic Na^+^/K^+^-ATPase is shown as a blue square. The Na^+^ taurocholate co-transporter (NTCP) at the basolateral membrane of hepatocytes mediates bile salt (BS) uptake, whereas a family of Na^+^-independent organic anion transporting polypeptides (OATPs) transports bile salts and non-bile salts organic anions (OA) both together. OC transporters (OCT) transport organic cations (OC) and certain organic anions (*e.g.* drugs) are transported by a group of OA transporters (OATs). The liver excrete bile salts through a canalicular BS export pump (BSEP) but Glutathione (GSH) and (non-BS) OAs are excreted via conjugate export pump in canalicles. In addition, there are export pumps in the canalicular membrane for organic cations (multidrug-resistance-associated protein 2; MRP2). [multidrug-resistance 1 (it is known as MDR1) gene product], phospholipids (PL) (it is known as MDR3), and sulfate conjugates such as breast cancer related protein( BCRP). There are twinned canalicular half-transporters -ABCG5 and ABCG8 -transport sterols into bile and play an important role in the regulation of biliary cholesterol secretion. MRP3·and MRP4 are expressed in the basolateral membrane domain of hepatocytes and when they accumulate in the cytoplasm facilitates biliary constituents extrusion. Bile duct epithelial cells contain the CFTR, which is a Cl- channel that drives bicarbonate (HCO3-) excretion through a Cl--HCO3- anion exchanger (AE2). In addition, bile duct epithelial cells contain the apical Na^+^-dependent BS transporter (ASBT) and MRP3 for reabsorption and cholehepatic cycling of BS. The familial intrahepatic cholestasis 1 (FIC1) protein (purple circle) is a P-type ATPase, its function is not yet understood well (it seems that it translocates aminophospholipids within the canalicular membrane). However, it seems it is a key element for bile formation because some mutations of these transporters were cleared in certain forms of genetic cholestasis (13, 17 and 20)

The main membrane transporters and molecular participants ascertain hepatic bile production largely characterized. Most of them have been cloned from both human and rodent tissues. Information on the localization, nomination and function of hepatobiliary transporters is indexed in Table 1. Some transporters play specific physiological roles in other tissues. The basic concepts of hepatic transporters involved in bile formation were reviewed previously (Figure 1) (13, 17 and 20). Below, we described the unique transport mechanism based upon the localization within the plasma membrane of liver cells.

*Mechanism of the hepatobiliary transport system*

The liver cells provide two polar surfaces that help for the reuptake of bile salts and help for the outflow of bile. The composition is further changed inside the bile canaliculi due to the apical and basolateral cholangiocytes transport system. Figure 1 illustrates the hepatocellular and bile duct transport system proteins. They promote the efflux and influx of endogenous and exogenous cholephilic substances (7) (Figure 1). 

*Biliary solutes and Sinusoidal uptake system*

The sinusoidal membrane of hepatocytes is embedded with certain proteins that act as a carrier molecule and assist BS and other lipid-soluble organic compounds in entering the liver. Both physiological substrates like bilirubin and other exogenous xenobiotic follow the same mechanism. The mechanism of Bile salt uptake can be either dependent on Na^+^ or may be independent. Two transporters, the Na^+^ taurocholate co-transporting polypeptide (NTCP) and other specific organic anion transporters, function in the process of bile formation. The NTCP is highly expressed in the basolateral surface of hepatic cells. It brings about the transportation of BS through the Na^+^-dependent mechanism into the hepatocyte (20). The membrane Na^+^ gradient of the liver cell controlled by the Na^+^/K^+^-ATPase helps NTCP transport BS mainly in the conjugated form (14, 20). The sinusoidal Na^+^ independent transportation of BS and organic anions is mediated by the organic anion transporting polypeptides (OATPs) system. The OATPs are the transporters with high affinity for a specific substrate and mediate uptake of unconjugated species of BS (21). In addition, due to the charge and structure variation of OATPs, a vast number of compounds like some bilirubin conjugates, thyroid hormones, steroids, drugs and other xenobiotics are transported (20, 21). 

Enormous OATPs have been determined both in rat and human beings. Organic anion transporting polypeptides (OATPs) are not only limited to hepatic tissues. They are also found in the intestine, kidney and brain (21). Some of the human OATPs are not similar to mouse and rat genes. However, those expressed in the liver have almost functions to mouse or rat OATPs*. Some of OATP members are involved in anion exchange which have decrease glutathione and bicarbonate, however, there are also observations of bi-directional transport (20). They help to exocytose potentially harmful substance (22). Recently some of the kidney organic anion transporters (OATs) and polyspecific organic cation transporters (OCTs) homologs are also found on the sinusoidal domain of hepatocytes, that contributes to the uptake of drug by the liver (23, 24).

*Biliary constituents and canalicular transport system*

Followed by intracellular transport, the biliary constituents are then secreted through the canalicular route within hepatocytes. This process is enhancing the bile flow and is important for the secretory ability of the liver. Drugs and xenobiotics are also excreted by this mechanism. ATP-binding cassette (ABC) transporters, which are unidirectional ATP-dependent export pumps, are indispensable in the process of bile formation (25). ABC transporters in the canaliculus membrane constitute of BS export pump (BSEP), which facilitates the secretion of BS into the canaliculus, multidrug-resistant-associated protein 2 (MRP2), which helps transport anionic conjugates and reduced glutathione, multidrug-resistant 1 (*MDR1*) gene product, which transport cationic compounds and steroids; and *MDR3* gene product, which translocate phospholipid. Inside the canalicular membrane, three additional ‘half ABC transporters’ exist, which have only six of the twelve trans-membrane domains of typical ABC transporters (25). These are twinned sterol half-transporters, ABCG5 and ABCG8. These have been suggested to perform a regulatory role in releasing of cholesterol into the bile (26). The breast cancer-related protein (BCRP), BCRP usually transports the sulfate conjugates (27). 

Amino phospholipids are transported along the canicular membrane of hepatocytes and cholangiocytes through P-type ATPase (familial intrahepatic cholestasis 1; FIC1) while carbonate is excreted through an anion exchanger into the bile (Table1). FIC1 may be associated with the dysregulation of the bile acid transport system or alteration in lipid composition within the canalicular membrane. However, more investigations are still needed to justify this assumption (13).

*Biliary constituents and Basolateral efflux*

A sinusoidal bile salt efflux is accomplished by mechanisms like OATPs or the ABC transport system presented within the basolateral membrane. MRP-3 and -4 transporters have several specific substrates, including sulfated and non-sulfated BS (25). A normal liver is usually unable to express a high level of MRP-3, but it can be induced (28). MRP-4 is liable to be expressed in the liver. It shows its activity by acting on ATP-dependent co-transporter and monoanionic BS, thereby reducing glutathione level and monoanionic BS (29). Biliary constituents follow an alternative way for its elimination by opening into the portal blood. This pathway is highly beneficial as it blocks the accumulation of toxic biliary constituents in the canalicular secretory pathway later which causes damage (see later).

*Cholangiocytes and associated transport systems*

Bile concentration can be significantly altered through a range of apical membrane transmembrane transport systems present on the cholangiocytes. Many efforts have been made to uncover the biology of cholangiocytes of rodents and human beings (13, 30). Various proteins that play a vital role in transportation has been discovered within the biliary epithelia. These transporters’ proteins are listed in Table 1.

**Table 1:** Localization and role of main membrane transporters relevant for bile formation (13, 17 and 20).

**Location**	**Transporter/gene nomenclaturec**	**Main Role**
Basolateral membrane of hepatocytes	OATs/SLC22A	Na^+^-independent transport of para-aminohippurate, salicylate, methotrexate and acetylsalicylate
	OCT/SLC22A	Hepatic uptake of hydrophilic organic cations. Essential components for drug transport.
	NTCPs/SLC10A1	Na^+^-dependent uptake of conjugated bile salt from portal blood.
	OATPs/SLC2IA	Na^+^-independent uptake of unconjugated bile salts and other organic anions.
	MRP 4/ABCC4	Glutathione efflux from hepatocytes into blood by co-transport with monoanionic bile salts. Possible an overflow pathway during cholestasis.
	MRP 3/ABCC3	Basolateral efflux of biliary ingredients including non-sulfated and sulfated bile salts.
Canalicular membrane of hepatocytes	BSEP/ABCB11	ATP-related bile salt transport into bile.
	MDR3/ABCB4	Essential components for biliary phospholipid secretion.
	MRP2/ABCC2	Transports bilirubin diglucuronide, sulfates, glutathione conjugates and numerous organic anions into bile in an ATP-dependent way.
	MDR1/ABCBI	ATP-related transfer of bulky organic cations into bile.
	ABCG5/ABCG8	Transport sterols into bile. They could also partly mediate biliary cholesterol secretion.
	BCRP/ABCG2	Cellular extrusion of sulfated conjugates.
	FIC1/ATP8B1	ATP-dependent aminophospholipid translocate. However, FIC1 role is not yet obviously clear.
	AE2/SLC4A2	Simplifies bicarbonate secretion into bile and involves in bile-salt-independent bile flow.
Apical membrane of cholangiocytes	ASBT/SLCI0A2	Essential for maintaining the enterohepatic circulation of bile salts

Abbreviations: ABC: ATP-binding cassette; ABST: apical Na^+^-dependent bile salt transporter; AE2: chloride-bicarbonate anion exchanger isoform 2; BCRP: breast cancer related protein; BSEP: bile salt export pump; FIC1: familial intrahepatic cholestasis 1; MDR: multidrug resistance; MRP: multidrug-resistance-associated protein; NTCP: Na^+^ taurocholate co-transporting polypeptide; OATs: Organic anion transporters; OATPs: organic anion transporting polypeptides; OCT: Organic cation transporter.

**Role of hepatocellular transporter systems in drug-induced cholestasis**

**Figure 2:**
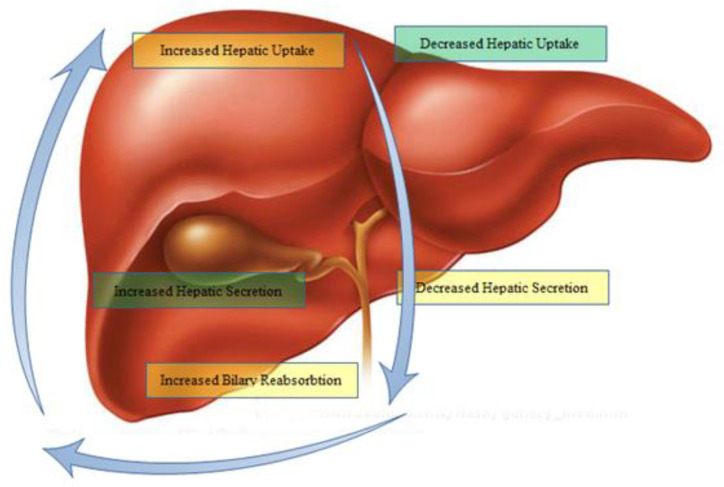
Transporter-associated process in the development of liver injury. Yellow and green fonts indicate possibly harmful or defensive transporter related mechanisms, respectively (7)

Animal models and *in-vitro *educations of drug-evoked liver injury expose several pump-associated processes that could contribute to liver injury (Figure 2). 

Drugs can induce liver injury through numerous distinct and overlying patho-mechanisms. Often, the precise molecular process of toxicity remains vague and, to some degree, unpredictable, especially for idiosyncratic responses (30). Drugs with cholestatic possibility require to be translocating into hepatocytes. Hepatic basolateral transport systems, like OATPs and NCTPs, might control drugs uptakes and their intracellular concentrations. As a result, these transporters can relate to drug-induced cholestasis (30). Indeed, OATPs are involved in the uptake of several drugs, such as antibiotics, bosentan, statins, and chemotherapeutic drugs (31) related to the cholestatic liver injury (Table 2). 

**Table 2:** Drugs related with induced cholestasis (91).

**Clinical signature**	**Drugs**
Vanishing bile duct syndrome	Amitriptyline, amoxicillin/clavulanic acid, azathioprine, barbiturates, carbamazepine, chlorpromazine, cimetidine, ciprofloxacin, clindamycin, diazepam, erythromycin, estradiol, flucloxacillin, glibenclamide, haloperidol, ibuprofen, imipramine, methyltestosterone, phenytoin, terbinafine, tetracyclines, trimethoprim sulfamethoxazole
Cholestatic hepatitis associated with bile duct injury	Amoxicillin-clavulanic acid, dextropropxyphene, flucoxacillin, pioglitazon
Cholestatic hepatitis	Acitretin, amoxicillin-clavulanic acid, atorvastatin, azathioprine, bupropion, celecoxib, carbamazepine, fenofibrate,chlorpromazine, macrolide antibiotics,gabapentin, glimepiride, isoniazid, itraconazole, metformin, methyldopa, captopril, ticlopidine, orlistat, propafenone, propylthiouracil, risperidone, terbinafine ,tricyclic antidepressants, troglitazone
Bland cholestasis	Anabolic steroids, azathioprine, cetirizine, glimepiride, cyclosporine A, tamoxifen, nevirapine

The suppressive action of drugs on the function of these basolateral transporters may be involved in the pathogenesis of cholestasis. Rifampin inhibits the human OATPs in the order of OATP8 > OATP-C > OATPA > OATP-B (31). There is no data for alteration of other basolateral transport system functions by a drug. However, it is estimated that blocking of uptake of liver toxicants by OATPs may preclude liver injury. For example, cyclosporine and rifampicin antibiotics as substrate and inhibitor of OATP1B3 respectively disable the ability of amanitin uptake in transected MDCKII cells (31). Therefore, pharmaceutical blocking of hepatotoxic entrance gates may be a promising therapeutic approach for drug induced hepatic liver injury. 

**Figure 3:**
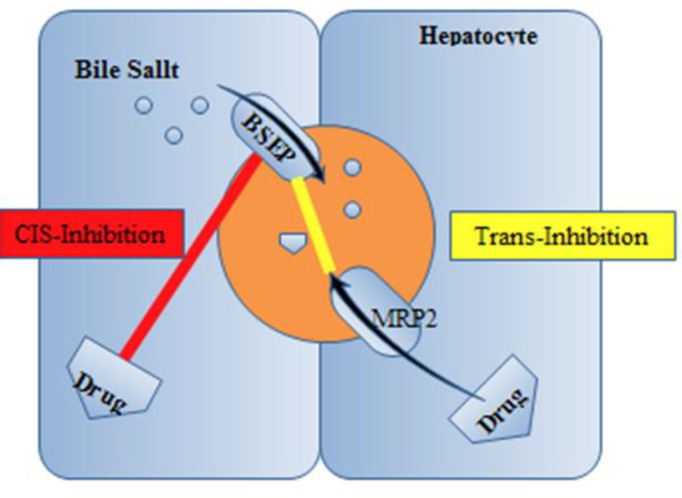
Inhibition of BSEP. Abbreviations: BSEP, bile salt export pump; MRP2, multidrug resistance–associated protein 2 (7)

Several drugs are supposed to induce cholestasis on the canalicular border via an inhibitory action on BSEP (Figure 3). 

*In-vitro* reports with rat Bsep exposed that cyclosporine, bosentan, rifampin, glibenclamide, and troglitazone prevent ATP-dependent taurocholate transport (32-34). While the competitive-based cis-inhibition presents the major route of BSEP suppression, some drugs such as 17 β-glucuronide and progesterone metabolites act through trans-inhibitory mechanisms promoted indirectly by Mrp2 into the bile ducts (32). Also, other drugs such as cyclosporine, verapamil, and vinblastine displayed *in-vitro *translocation by MDR3, which could cause concentration-dependent prevention of phospholipid flippase activity (35). A supplementary patho-mechanism of cholestatic cell injury was lately suggested by Fouassier *et al.*, who examined the role of bosentan on bile formation by Mrp2 (35). It can be displayed that bosentan activates and meaningfully enhances Mrp2-mediated releasing of bilirubin and salt–independent bile flow. Mrp2-deficient TR−rats did not show a defect in the biliary lipid component. This phenomenon indicates that the function of the canalicular membrane in transporting organic anions is pivotal for developing the uncoupling outcome. Therefore, Mrp2-evoked cholestasis may reduce the bile salt content of bile canaliculi under the concentration essential for the dissolving of phosphatidylcholine and cholesterol. Therefore, reduced biliary phospholipid excretion does not essentially mean a fault in the canalicular phospholipid flippase MDR3; however, it may be clarified by a physicochemical imbalance in bile formation. A new study proposes the participation of the prevention of MDR3 in itraconazole-evoked cholestasis. *In-vitro *examines displayed that itraconazole restrained MDR3- mediated biliary phospholipids excretion without meaningfully influencing BSEP-mediated transport of taurocholate (probe substrate for biliary acid transport). In rats, the biliary excretion of phospholipids was obviously decreased after itraconazole administration. Whereas inadequate evidence is accessible about the interaction with MDR3 by cholestatic drugs, the probability of MDR3 prevention, plus BSEP inhibition, explains a better understanding of the process of drug-related cholestasis (36).

In addition to these hepatic causes of drug poisoning, it is possible that drug uptake by cholangiocytes and cholehepatic shunting involves in the progress of hepatic injury. For example, the nonsteroidal anti-inflammatory drug sulindac was exposed to tempt hypercholeresis in animal models, a phenomenon described by cholehepatic shunting (37). The subsequent hepatic deposition of sulindac and the prevention of canalicular bile salt transportation may donate the capacity of this drug to create cholestasis (38, 39).

**Present methods for recognizing and avoiding drug-induced cholestasis**

DIC is of specific alarm because it is the main reason for removal through drug development (40) and has been accountable frequently for withdrawals of drugs from the market (41, 42). Additionally, hepatotoxicity is the very frequent single side drug effect leading to refusal for drug approval and regulatory actions by the US FDA (43). So, it is vital to expand our information and description of DIC and elaborate better forecast models to recognize and mitigate potential agent-connected safety problems. These attempts will consequence in harmless drugs and more effective drug discovery and development procedure. Table 3 lists a summary of the present models for examination of a drug-induced cholestatic potential.

**Table 3:** Present models for the recognition of drugs with cholestatic capability (107, 108).

*In-vivo *based-models	Bile duct-cannulated rodent models Hepatobiliary transporters rodent wild-type and knockout models Clinical studies Phase I, II, III and IV Large prospective clinical trials
*In-vitro *models	Vesicular transport (VT) assay using membrane vesicles, transfected cell lines or suspended hepatocytes ATPase assay using transfected cell lines or suspended hepatocytes *In-vitro *biliary clearance technique using accumulation studies in sandwich-cultured hepatocytes (human and preclinical species) Sandwich-cultured hepatocyte uptake and efflux assay
*In-silico *models	Structure- activity relationship (SAR) and quantitative SAR modeling 3D-pharmacophore modeling Chemical category approach Analogue/read-across approach Drug elimination pathways

*In-vitro Methods Used To Determine In-vitro Biliary Excretion*

*In-vitro *methods have been recognized to examine the possibility for drugs to interfere with bile salt pumps. Types of *in-vitro *systems appropriate to evaluate transporter function are either membrane-based or cell-based. Preclinical *in-vitro *models can support more effective progress of safer drugs by recognizing hepatotoxic possibility through the initial phases of the procedure. Additionally, when a hepato-toxicant is anticipated, *in-vitro *approach systems can aid in the perception of pathogenic pathways and may support the design and analysis of alternative agents to avoid the cholestasis potential. Hypothetically, a beneficial *in-vitro *method is the one similar to *in-vivo *conditions leading to cholestasis and procreate intended hepatic functions (44, 45).

Currently is possible to assess interactions underlying drug up taking and bile and other cellular components secreting pathways through numerous *in-vitro *methods. Using these methods is possible to scrutinize the cell functions as a whole or break down the functions in detail at the levels of intracellular membranes. For example, it is practical to study vesicular membrane proteins by transfecting a particular transporter using cell lines. Such methods support human and animal *in-vivo *investigations and permit a description of transport procedures at the molecular step. Unluckily, the outcomes acquired with *in-vitro *studies are problematic to compare to human *in-vivo *results.

*Membrane-Based Assays of ABC Transporters*

Membranes isolated from cells expressing transporters have been extensively applied to analyze the property of ABC efflux transporter and recognize their substrates or inhibitors. Presently, there are two membrane-based tests can be used for predicting drug-transporter interaction in regard to cholestasis: the ATPase test and the membrane vesicular transport test. Compared to the cell-based analyze, the membrane-based test has numerous benefits involving: (a) the test can be employed to describe the consequence of a xenobiotic on one particular efflux transporter; (b) the test can be simply utilized in a high throughput style, (c) the maintenance of membranes are after preparation; and (d) the examine is comfortable to direct.

**Assay Types**

*ATPase Assays*

ATPase tests are based on ABC-related ion-translocation against a concentration gradient using energy resulting from ATP hydrolysis. It is an easy and applied method that can be operated in high-throughput tests to monitor for agents that interfere with some ABC pumps. Enhancement in transporter basal ATPase function indicates a probable substrate, though some gradually transported substrates do not display a significant larger increase in transporter function than the baseline (45).

By introducing BSEP, human-mouse ortholog, into baculovirus-infected cells derived from insect (Sf9) system, and the impacts of membrane cholesterol content was assessed on membrane transporting activity. The pump performance of cholesterol-loaded mouse Bsep-HAM-Sf9 vesicles was investigated in a vesicular pump analysis with taurochenodeoxycholate (TCDC), a recognized BSEP probe. Mouse Bsep passages TCDC to a great degree that can be profoundly identified in the ATPase evaluation. Cholesterol upload of the Sf9 membrane triggers both TCDC passage and TCDC-induced ATPase stimulation. It was shown that a meaningful grade order correlation was discovered between IC_50_ levels calculated in the TCDC-activated mBsep ATPase test and the human BSEP vesicular passage assay employing taurocholate (TC) as probe compound. This updated type of mouse, Bsep-HAM ATPase test, is a convenient, sensitive, nonradioactive technique for initial high-throughput examination of drugs with BSEP-associated cholestatic possibility. It can accompany the human BSEP-related taurocholate vesicular pump inhibition test (46).

*Membrane Vesicular Transport Studies*

The membrane vesicular transport test is another high throughput examination that can be employed to recognize substrates and/or inhibitors of efflux transporter, such as P-gp, MRP, BCRP or BSEP, and influx pumps, such as NTCP. The membrane vesicles can be obtained not only from over-expressed cells or transporter-transfected but also from luminal and abluminal membranes of the brain, the brush border membrane of kidney, intestine and choroids plexus; hepatic sinusoidal and canalicular membranes (47, 48). Because several different transporters express in membrane vesicles acquired from tissues, their value for estimating the interaction of a given transporter with a given agent is accompanied by limitations. The propriety of these tissue membrane vesicles can be assessed by improving the virtual activity of marker enzymes for the target plasma membranes (47). The isolation and refining of membrane vesicles are long-lasting and technologically contesting. However, when the membrane vesicles are created and illustrated, they do not require preservation and can be kept for many months at < -80 ºC. The marketable obtainability of the efflux pump expressing membrane vesicles produces this test practicable and capable to be operated regularly. The majority of ABC pumps and some SLC transporters can be examined by means of membrane vesicle experiments. Because NTCP is a sodium related pump, the NTCP function can be evaluated by quantifying the alteration of drug concentration in the right-side-out membrane vesicles (the pump is in the same direction as it is in the cells) in the existence and deficiency of Na^+^ buffer. The amount of Km (the concentration at which half the maximal uptake happens) of taurocholate for NTCP was measured to be 45 µM utilizing membrane vesicles of rat liver sinusoidal (SMV) (49) which is equal to the Km (32~41 µM) acquired from sandwich cultured rat hepatocytes (50), representing that liver SMV is a valuable device to assess the activity of NTCP. Using a quick purification method, the membrane vesicles can be accumulated on a filter film, and the substrate imprisoned within the vesicles can be measured by a fluorescence detector, LC/MS/MS, or liquid scintillation counting (51). As a practical test, the membrane vesicle transport assay has been applied to examine binding sites (52, 53), interspecies variations in pump function (53, 54), polymorphisms in transporter performance (55, 56) and substrate or inhibitor selectivity for a provided efflux transporter (57, 58).

Furthermore to the abovementioned purposes, cell membrane vesicles or liver cell CMVs expressing BSEP have been employed to identify the cholestatic possibility of BSEP inhibitors and have been accepted to monitor the agents for the capability to produce cholestasis in the liver (34). Membrane vesicles obtained from primary liver cells have clear benefits over transfected systems: the natural membrane situation is in position through separation from the other cellular elements, and the hepatic transport mechanisms exist completely. Vesicular membranes can be purified, kept frozen and employed as required; but, the preparation of basolateral and canalicular portions is labor exhaustive, and absolute purity is never attained.

Membranes attained from baculovirus-transfected insect cells overexpressing BSEP/Bsep can be utilized in both vesicular transport and the ATPase tests, but liver canalicular membrane vesicles, achieved from hepatocytes, can only be employed in VT tests (45). Experiments based on vesicular transport tests display that BSEP function depends on the cholesterol concentration of the surrounding membrane. An enhancement in the cholesterol amount of vesicles, particularly those that originated from the canalicular liver membrane, has been presented to enhance taurocholate influx activity (59). But the vesicular transport test is extremely specialized for measuring interference in BSEP function unrelated to other transporter systems (for example, the NTCP). This restriction may delay the detection of possible hepatic metabolism and toxic metabolites (45).

*Cell-Based Assays*

The majority of cell-based investigations are practical transporter tests. Because of the intact cell organization, cell-based tests may deliver more definitive evidence about the interaction between drugs and pump proteins and can be used to evaluate kinetic constants, such as Km and V max for substrates in addition to Ki or IC_50_ for inhibitors. These tests can also predict DILC that can happen in the clinic. With mechanization and cell culture in a multi-well plate, cell-based tests can be adjusted to a high throughput manner. Numerous disadvantages of cell-based tests include: (a) Expression of several pumps in a specific cell line even lines that have been engineered to express an assumed transporter; (b) the expression degree of translocators proteins alters with culture circumstances and quantity of cell passages; (c) the cells require to be kept under specific condition before use. This test is more labor-intensive and time-consuming than the ATPase test and membrane vesicular transport experiments. 

**Commonly Used Cell Systems**

*Sandwich-Cultured Hepatocytes*

**Figure 4:**
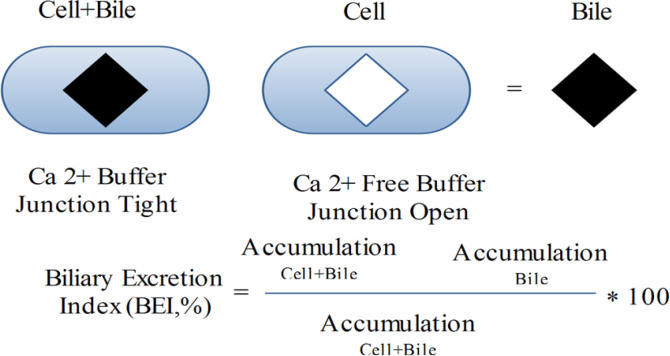
Working model of sandwich cultured in liver cell (58, 109).

Sandwich-cultured hepatocytes preserve the hepatocyte construction, such as tight junctions, canalicular biliary system, and practical pumps. As a consequence of these structures, the sandwich-cultured liver cells have developed a useful system to assess both uptake and efflux hepatobiliary pumps. Because diminution of Ca^2+^ is able to open tight junctions, concentrations of compounds in hepatocytes and bile ducts, or in hepatocytes is determined in Ca^2+^ and Ca^2+^ free buffer, correspondingly (58) (Figure 4). 

Efflux transporters, such as P-gp and MRP2, have been established in the cytoplasm of newly separated hepatocytes. In sandwich-cultured rat or human liver cells, repolarization of MRP2 and P-gp in canalicular membranes was detected by observing the colocalization of these pumps with dipeptidyl peptidase IV (DPPIV), an indicator protein of canalicular membranes (60). The activity of Pgp in sandwich cultured hepatocytes was exhibited by dynamic biliary excretion of [3H]-digoxin, rhodamine 123 (61) and [3H]-DPDPE (60). Oatp1, Oatp2 (OATP2 and OATP8 for human liver cells) and NTCP were expressed in sandwich cultured rat or human hepatocytes and their activities preserved during the culture phase (60). In a Na^+^-related way, taurocholate, an endogenous bile salt, was taken up mainly by NTCP into the sandwich cultured hepatocytes (50) and energetically discharged into the bile canalicular system by BSEP (62). Bosentan (an oral endothelin-1 receptor antagonist) can produce liver cholestasis. This drug could block taurocholate biliary excretion in sandwich cultured rat hepatocytes by blocking both NTCP and BSEP. So, sandwich cultured hepatocytes can be employed to assess hepato-biliary transporter-related DIC (62). The outcomes acquired from one donor’s liver cells might not be extended to the entire human population due to the probable polymorphisms. Another drawback of liver cell investigation is the obtainability of the plateable cryopreserved human liver cells since the transporter experiments need the construction of a canalicular biliary system. Employing rat and human Sandwich-cultured hepatocyte, a good estimation of biliary clearance rate *in-vivo *for angiotensin II receptor blockers, b-lactam antibiotics, and statins could be foreseen by *in-vitro *intrinsic biliary clearance (63, 64). A Human Sandwich-cultured hepatocyte was applied to calculate the interaction in BSEP function of numerous drugs stated to cause cholestasis. Cyclosporine A, glibenclamide, bosentan, erythromycin estolate, CI-1034 and troleandomycin powerfully blocked [3H] taurocholic acid efflux, though macrolide antibiotics were much less strong blockers. The potency blocking of taurocholic acid efflux is linked with the occurrence of clinical hepatotoxicity, proposing that *in-vitro *inhibition may be predictive of cholestatic possibility *in-vivo *(65).

*Suspended Hepatocytes*

The primary liver cells can be alive for several hours in suspension if supported with suitable culture medium and oxygen. Clarifying metabolic pathways of drugs in hepatocytes can be achieved by preserving both rat and human cells in freeze conditions (66, 67). These cells are alive and valuable resources for ex vivo examination of membrane transport systems in hepatocytes. Furthermore, these cell are applicable for blocking investigations that can be functioned with this process. However, because of the restricted sustainability of these cells, it is not probable to analyze stimulation processes (62, 68 and 69).

Moreover, internalization of canalicular ion-translocator pump through collagenase perfusion restricts the application of suspended hepatocytes to foresee a general value for biliary clearance when pumping into bile is valuable. Recently isolated or cryopreserved liver cell suspension can be applied for hepatobiliary uptake pump but not efflux pump investigations (60, 70, 71) because of internalization of efflux pumps after isolation (59). Sugiyama’s team scrutinized NTCP-related uptake of thaurocholate and OATP-related uptake of estradiol-17-glucuronide (E17G) in the human liver cell prior and following cryopreservation. They showed that the cryopreservation system could alter the function of uptake pumps (71).

*Transporter-transfected cell lines*

Other valuable *in-vitro *methods to examine BSEP/Bsep-related taurocholate translocation are MDCKII (Madin-Darby canine kidney type II) or LLC-PK1 (pig kidney proximal tubule) cell lines, which can be transfected with NTCP/Ntcp, BSEP/Bsep and/or MDR3 (36, 72, 73), as these transporters express endogenously in low levels. 

Cells that have been permanently or transitorily transfected with efflux or uptake pumps have been investigated to examine pump activity, substrate or blocker recognition, species alteration, and polymorphisms. Uptake and efflux transporters have been effectively transfected to numerous cell lines such as HEK, MDCKII, LLC-PK1, Hela, and HepG2 cells. All of these transfectants can be utilized for uptake and cell sustainability tests, though MDCKII and LLC-PK1 transfectants can also be applied for bidirectional pumping analyses. The polarized MDCKII cells have been applied widely to express numerous mixtures of recombinant import and export pumps. Initially, In 1989, Pastan *et al.* produced MDR1- transfected MDCKII cells. These cells have been extensively applied to illustrate the substrates and blockers of P-gp, predict the penetrability of P-gp substrates through blood-brain barrier (61, 74). Cui and his colleagues first established OATP8 and MRP2 double transfected MDCKII cells to assess hepatic pump function (61). Next, the similar team produced OATP-B, OATP2, OATP8 and MRP2 quadruple-transfected MDCKII cells for the description pump-related process of drug transfer bile and uptake into hepatocytes (75). The immunofluorescence microscopy experiments displayed the MRP2 (efflux pump) is located on the apical border of MDCKII cell membranes, and the other pumps are expressed on the basolateral border. In the quadruple-transfected cells, the intracellular content and trans-cellular transfer degree of organic anions, including cholecystokinin peptide (CCK-8), bromosulfophthalein (BSP), and estrone sulfate were higher compare to the double transfectant. In the quadruple-transfectant substrates for uptake or vectorial transport, identification is facilitated; however, these may be ignored in the tests with double or single transfected cells. For instance, Sugiyama and his colleagues created numerous double transfected MDCKII cells expressing OATP2/MRP2, OATP2/BCRP and OATP2/P-gp attempt to show the relative involvement of BCRP, MRP2 and P-gp to the translocation of organic anions through the bile canalicular membrane (76). Utilizing these three double transfectant, the main pumps contributed in the biliary clearance of pravastatin and estradiol17-glucuronide was recognized as MRP2. Both MRP2and P-gp were contributed in the excretion of cerivastatin and BCRP was the main pump that contributed in the secretion of estrone sulfate. These instances exhibit that the double or quadruple transfected cells are useful tests for the examination of the function of hepatobiliary pumps in addition to sandwich-cultured hepatocytes.

The advantages of this model are; transfection of distinct transporter, cell line grown on a permeable membrane, and applied to analysis the trans-cellular movement of certain substrates (77). On the other hand, they have a deficiency in the total complement of pumps, enzymes, and cofactors existent in liver cells and pumps expression content may not be similar to those of liver cells *in-vivo*. These problems must be evaluated in outcomes explanation and when extrapolating to *in-vivo *biliary clearance parameter. However, transfected methods are easy to apply and more simply accessible than primary human hepatocytes and are valuable as a high-throughput screening method.

*Use of in-vitro assay protocols for evaluation DIC*

BSEP-expressing membrane vesicles test were effectively applied to assess a vast selection of standard agents interfering with BSEP activity and their association with clinical DIC (78, 79). Regarding the association between BSEP inhibition potency (defined by IC_50_) and clinical DILI, Morgan *et al.*’s experiment was accepted for more than 200 drugs (79). 

One collection of medications was arbitrarily chosen, while others were related to BSEP-related liver damage in humans. Approximately 75% of tested drugs presented unimportant or no interaction (IC_50_ ≥ 133 µM) with BSEP pump in association with their liver dangers problems in humans and/or exhibited that the process of liver damages was unrelated to BSEP. High-level potency of BSEP blocking (IC_50_ ≤ 25 µM) was observed in 60% of the examined drugs. These drugs include endothelin antagonists, thiazolidinediones, sulfonylureas, protease inhibitors and antibiotics, which are related to DILI in humans that the possible function of BSEP interaction in the liver damage has been formerly defined. 

Furthermore, this report supplied novel suggestions of the involving process of liver problems related to numerous kinase inhibitors. The chemotherapeutic agents, sorafenib, imatinib, pazopanib, lapatinib, and gefitinib blocked BSEP with comparatively high potencies (79). In another examination, managed by Dawson *et al.* (78), 85 drugs were examined to block hBSEP/rBsep. Sixty-four of these drugs induce clinical DILI classified based on their form of liver damage: hepatocellular, cholestatic or mixed cholestatic/hepatocellular. The occurrence of *in-vivo* mixed, and cholestatic liver damage in agents with *in-vitro* hBSEP inhibition IC_50_ lower than 300 µM were greater than agents with *in-vitro* hBSEP inhibition IC_50_ greater than 300 µM which produced hepatocellular or no DILI. These researchers proposed an *in-vitro *IC_50_ lower than 300 µM can be exploited to characterize an agent with important cholestatic capability *in-vivo*. Concerning the physicochemical possessions of the drugs discovered that all agents with hBSEP IC_50_ 300 µM had hydrophobicity partition coefficient (ClogP) 1.5, molecular weight (MW) 250, and nonpolar association surface area 180 A˚ (see section 4.5) (78).

It is prominent that 38% of drugs accompanying DIC in humans did not block hBSEP *in-vitro *(IC_50_ values 1000 µM). It displays that inhibition of the BSEP is one of the processes that might induce DIC (78). The creation of reactive metabolite has also been involved in DILI, and in some examples, metabolites can display stronger pump inhibition than the initial drug (34). Dawson *et al.* examination (78) performed only on the drugs and not on the metabolites.

Lack of information about potential metabolic drugs is a restriction of the vesicle transport test. The investigation delivered an initial proof of *in-vitro *hBSEP blocking for numerous market drugs that are stated to induce DILI: dicloxacillin, glafenine, alpidem, bezafibrate, flucloxacillin, ticlopidine acitretin, and leflunomide (78). Inhibition of the canalicular efflux pump may involve patho-mechanism of the liver damages related to these agents.

Generally, the results of these two comprehensive reports reveal that blocking of hBSEP/rBsep associates with the tendency of many drugs to induce DIC in humans. *In-vitro*, high-throughput models deliver a validated prediction tool for DIC that goes hidden through preclinical assessment.

Sf9 cell-derived vesicles and SCH were applied to examine the process of hepatotoxicity of anti-retrovirals. In the human systems, the grade inhibition of BSEP was as follows: ritonavir > saquinavir > efavirenz, nevirapine = 0 though this grading was not capable of predicting the hepatotoxic clinical property of these drugs (nevirapine > ritonavir > saquinavir = efavirenz = 0). The results propose that blocking of BSEP by anti-retrovirals is not the important process for their hepatic side effect. Any possible incensement of bile acids because of BSEP inhibition seems to be stabilized by simultaneous blocking of the uptake pumps NTCP and OATP. Ritonavir, saquinavir and efavirenz blocked taurocholate uptake carriage in recombinant NTCP and rat Ntcp with IC_50_ constants varying from 2.1 to 43 µM (80).

*In-vivo models*

Many hepatotoxicants go unobserved in experimental toxicology investigations that apply animal methods because of species specificity problems. Species alterations in pump expression, physiological property, substrate affinity, compensatory process and metabolic ways can result in noticeable variations in tolerance to cholestatic agents (72).

The evaluation of the similarities and variances of the molecular features and performance of BSEP are regularly applied in experimental species, such as rodents and dogs, may support the clarification of the species inconsistency on predicting DIC in humans (30). Even though BSEP is an extremely preserved 1321-amino-acid protein, the mice ortholog likeness with human BSEP and rat Bsep, are 89 and 94% correspondingly (81), and the dog Bsep displays 89 and 80% similarity with human BSEP and rat Bsep, correspondingly (82); species alterations because of changes in the pump function were established. 

Significant alterations in BSEP inhibition potency for drugs with potential cholestasis were detected among species. For instance, IC_50_ levels for cyclosporine A, glibenclamide and troglitazone might vary in nearly one order of amount from human BSEP and mouse Bsep (46, 83). Similarly, a species variance in pravastatin affinity was detected. The IC_50_ of pravastatin for human BSEP, dog Bsep and rat Bsep were 240, 441, and 1000 µM, correspondingly (82). In examining 85 authorized drugs, there was a powerful association between potency levels for inhibition of hBSEP and rBsep; however, 14% of the examined agents showed above two-fold lesser levels for human pump IC_50_ than the rat orthologs. The greatest noticeable potency alterations were detected with drugs that are stated to induce cholestatic damage: ketoconazole (hBSEP IC_50_ fivefold lesser than rBsep), nefazodone (four-fold), pioglitazone (seven fold) and rosiglitazone (six-fold) (78).

Species variances in the affinity of substrate for MRP2 were also established. Mycophenolic acid blocked MRP2 function with a 3.6-fold greater potency in rats than in humans (84). Also, species variances in the expression of specific transporters may occur. For instance, it was reported that rat hepatic Mrp2 expression is 10-fold greater than humans (85). 

Variance in the blocking of uptake pumps among species was discovered as a description of the rats resistance to bosentan-stimulated hepatotoxicity detected in humans. Bosentan was understood to be a strong blocker of Ntcp than NTCP, as established by 6- and 30-fold alterations on IC_50_ levels of sodium-related taurocholate uptake in rats against human suspended hepatocytes and NTCP-/Ntcp-transfected human embryonic kidney (HEK) cells, respectively. So, special inhibition of rat Ntcp using bosentan must cause less intracellular concentration of bile acids in rats despite the blocking of Bsep. The bile salt taurocholate disposition extremely varies in human and rat liver cells. In human Sandwich-cultured hepatocytes, about 30% of taurocholate trapped in the cells, but it was only 7% in rat cells. 

In human Sandwich-cultured hepatocytes, canalicular and basolateral efflux pumps are involved similarly in the excretion of taurocholate, but in rat cells, the role of the basolateral efflux pump is more considerable (86). The blocking of BSEP/Bsep significantly caused greater intracellular accumulation of taurocholate in human Sandwich-cultured than in rat cells. The maximum intracellular concentration of taurocholate was detected with cyclosporine A and glibenclamide, which blocked the bi-directional translocation of taurocholate. These consequences propose that the greater percentage of basolateral bile salt efflux is an extra defensive process in cholestasis (86).

Another description for *in-vivo *species variances is a compensatory up-regulation of bile salt efflux pump. Intrahepatic cholestasis was seen in Bsep (bsep-/-) deficient mice (87), despite the lesser severity in humans with PFIC2 polymorphism (88). The greater expression content of Mdr1a,b in the apical membrane of liver cells may be described as these variations (88, 89). The compensatory function theory was established with a triple knockout (mdr1a-/-, 1b-/-, bsep-/-) animal that showed a more severe phenotype than bsep-/- mice: flaccid gallbladder, decreased bile creation, jaundice, and enhanced mortality (90). All the species differences as mentioned above may deliver rational descriptions to hepatotoxic potential inconsistencies among species and why experimental transport results suggest inadequate predictability of liver problems in humans. Species alterations must be considered precisely to explain the results and translate preclinical results to the clinic.

*In-silico methods*

The use of computational modeling systems to increase understanding of transporter-drug interactions is developing progressively. Obtainable datasets can be employed to construct *in-silico *models established on the drugs’ structural properties, and these systems can be applied to estimate drug influences on bile acid metabolism. When outsized datasets are obtainable and *in-silico *models are properly authenticated, they can decrease the financial problem in initial drug discovery and development by restricting the necessity for comprehensive laboratory investigations. The 3D constructions of membrane pump proteins remain scarce. So present computational pump surveys depend on the sequence of investigational calculated interactions of membrane transporters with small molecules and utilize statistical knowledge methods, for example, ligand-based pharmacophore construction and quantitative structure-activity relationships (QSAR). Obtainable computational models of bile acid pumps (*i.e*., MRP2, MRP3, MRP4, NTCP, BSEP, OATPs, ASBT) were reviewed previously (91). Due to the excessive heterogeneity in investigational findings (*e.g.*, from varied test categories, experiment concentrations, and investigational situations), very computational reports exhibit classification methods (*e.g.*, non-inhibitors *vs.* inhibitors). Warner *et al.* (92) described the categorization of BSEP blockers (described by an IC_50_ of 300 µM) by a recursive partitioning QSAR for over 600 agents through molecular descriptors as covariates. A novel QSAR system founded on ‘chemical fragmentation codes’ (*i.e*., called fragment of a chemical construction), effectively established by Saito *et al.* (93), permitted the recognition of specific chemical moieties strictly related to the blocking of BSEP pump function. The consequences of the QSAR report proposed that an ester or thioester group attached to a carbon of heterocyclic ring(s) is an essential chemical agent for the interaction with BSEP. Also, a carbocyclic system with at least one aromatic ring and mononuclear heterocycles definitely participated in the blockage of BSEP (93). Numerous computational algorithms have been provided to form a BSEP inhibition model for a group of 624 chemically various agents (92).

Numerous studies showed that QSAR models could be used towards prediction of MRP2 inhibition potency (91) by linear (*e.g*., discriminant study and partial least squares regression) and nonlinear modeling approaches [k nearest neighbors, random forest, support vector machine (SVM)]. The correctness of these systems on the results assessed by examination sets varied from 70% to 90%. Also, it was established a QSAR approach for 25 methotrexate analogs in regard to binding affinity to rat Mrp2 in addition to a pharmacophore for their binding model (94). In a novel experiment, Akanuma *et al.* evaluated the structural function of the MRP4 pump for numerous sets of β-lactam antibiotics (95). Sedykh *et al.* and Zheng *et al.* stated categorization QSARs of ASBT blockers were established on 10 and 100 µM potency levels correspondingly (96, 97). Recently, it was established a 3D-pharmacophore system based on five NTCP substrates, which were then used to evaluate large numbers of chemical agents (98).

The main restriction to evaluate the accuracy of *in-silico *method to predict drug interactions with bile acid pumps is the small datasets. Protein pumps conformational flexibility, substrate selectivity, investigational tests restrictions need a complete range of construction chemicals for appropriate statistical knowledge. Also large sample size is essential to achieve practical comprehensive outcomes (91). 

**Conclusion**

Impaired bile acid homeostasis is an essential process of drug-induced cholestasis. To predict the cholestatic capacity of agents precisely, perception of the interaction between drugs and various mechanisms contributed to bile acid homeostasis is essential. Drug-induced damage to hepatobiliary pumps is the main reason for cholestasis. The instances defined in this review focus on the methods pertaining to the pump’s activity, particularly vesicle membranes and sandwich-cultured hepatocytes through drug development. Additionally, novel progressions in cellular systems such as provoked pluripotent stem cells hold the potential to analyze the patho-mechanisms of DILI (99).

Among the possible pathogenetic processes of drug-induced cholestasis, the blocking of BSEP by agents is satisfactorily recognized (78). Interfering of a novel agent with BSEP transport function does not essentially indicate that it will be ignored from clinical progress, but investigational strategies should be precisely followed up, and liver performance during the clinical study has to be screened.

The alignment of *in-vitro *monitoring for transport interaction and *in-silico *predicting systems is a price- and time-effective plan. Novel *in-silico *methods displays potential cholestatic drugs. For instance, through a three-dimensional (3D)-pharmacophore approach using a library of 10 million agents can find those with potential NTCP inhibitory action. Developments of *in-vitro *and *In-vivo *investigations were directed to authenticate the function of the virtual monitoring hits. Consequent SAR examination showed key molecular characteristics for effective NTCP interaction (98). Also, molecular physicochemical features, particularly lipophilicity and MW, and metabolic pathways monitor the possibility of liver injury (79, 100).

Moreover, stem cells are valuable since they can demonstrate the patho-mechanisms of DILI, including DIC. The new group of stimulated pluripotent stem cells from somatic cells (100) differentiate into human hepatocyte in this subject. Human-induced pluripotent stem cells can be differentiated towards the hepatic lineage which presents possibilities for improving research on liver diseases and drug development (101, 102). Per se, they can substitute liver cell lines in drug toxicity evaluation tests (103), also delivering a chance to recognize potential properties of novel drug candidates on particular disorders (102). Also, they permit evaluating the influence of a genetic polymorphism on systems contributed in drug metabolism and translocation and its connection with drug side effects (104).

Although experimental animal models supported developing our knowledge of the function of hepatobiliary pumps in drug-induced cholestasis; now, they present restricted predictability of liver problems in humans. Species variations should be evaluated carefully to explain the results and interpret experimental discoveries to the clinic. The new development in the subject of genomics systems exhibits a capacity to resolve species-specific variations in the extrapolation of clinical toxicity of possible agents using experimental species. For instance, a toxicogenomics-based strategy effectively established that the recognized species inconsistency in the toxicity of coumarin could be clarified using wide-ranging fluctuations in gene expression in the rat *in-vivo *and *in-vitro *and human *in-vitro *methods (105).

Large development has been achieved in clarifying the role of genetics in verifying an individual’s tendency to progress liver disorders. Although the effect of polymorphic hepatobiliary efflux pump in the augmented risk of obtained cholestasis is now well recognized, additional investigation is required to describe the role of the genetic changeability of the hepatobiliary transporters as a vulnerability factor (106).

So the genotypes recognized may be valuable in experiments of the mechanisms of drug-induced cholestasis, may have prognostic worth and may supply a diagnostic approach in the clinic.
